# Assessing the prognostic value of respiratory oscillometry in patients with difficult-to-treat asthma

**DOI:** 10.1038/s41598-023-29672-z

**Published:** 2023-02-11

**Authors:** Yi-Luen Shen, Yi-An Hsieh, Yu-Ming Huang, Yi-Hao Peng, Ling-I Chen, Fang-Chuan Dai, Yu-Sheng Lin, Chien-Wen Huang

**Affiliations:** 1 Division of Chest Medicine, Department of Internal Medicine, Asia University Hospital, No. 222, Fuxin Rd., Wufeng Dist., Taichung City, 41354 Taiwan, ROC; 2 Department of Respiratory Therapy, Asia University Hospital, Taichung, Taiwan, ROC; 3 Department of Medical Laboratory Science and Biotechnology, College of Medical and Health Science, Asia University, Taichung, Taiwan, ROC

**Keywords:** Asthma, Prognostic markers

## Abstract

Respiratory oscillometry is widely explored in asthma management; however, there is currently no consensus on its routine work-up in patients with difficult-to-treat asthma. We conducted a retrospective, cross-sectional study involving patients with difficult-to-treat asthma at Asia University Hospital between January 2017 and October 2020. We aimed to correlate clinical significance of respiratory oscillometry and asthma treatment outcomes including symptoms control and exacerbation in patients with difficult-to-treat asthma. Among the 69 patients enrolled in the study, a total of 26.1% of the patients experienced at least one severe or two moderate exacerbations. Patients with ACT < 20 presented a higher prevalence of higher frequency-dependent resistance (FDR; the difference in resistance at 5 Hz and 20 Hz) and frequency of resonance (Fres) than those with ACT ≥ 20. In the multivariable analysis, comorbidities, COPD or allergic rhinitis, and FDR were independent factors in increasing the odds ratio in poorly controlled asthma. (FDR ≥ 0.10 vs. < 0.10, adjusted ORR = 5.05, *P* = 0.037) There was a higher proportion of frequent exacerbations in patients with higher FDR (FDR ≥ 0.10 vs. < 0.10 = 30.0%:20.7%), but IOS parameters failed to predict frequent exacerbations on further analysis. FDR may be a potential clinical parameter for predicting symptom control in patients with difficult-to-treat asthma.

## Introduction

Asthma is characterized by chronic airway inflammation, which results in airway hyperresponsiveness, obstruction, mucus hyperproduction, and airway remodeling^1–3^. The prevalence and economic burden of asthma increase yearly, especially in poorly-controlled asthma^4–6^. The definition of poorly-controlled asthma is re-aligned in 2019 Global Initiative For Asthma (GINA) guideline, which conceptualizes “Uncontrolled asthma,” “Difficult-to-treat asthma,” and “Severe asthma”^7^. Once patients are diagnosed with difficult-to-treat asthma, modifiable factors such as adherence, inhaler technique, medication side effects, and comorbidities should be reviewed and corrected. Patients with persistent uncontrolled asthma after maximal optimal therapy and treatment of contributory factors within the window of time were diagnosed with severe asthma. The clinicians heavily use patients' self-reported symptoms in the clinical assessment of asthma control and treatment modifications^8^. Pulmonary function also provides clinicians with another aspect of disease control; however, the discrepancy between asthma control scores and pulmonary function tests existed in previous studies^9,10^. Spirometry is not well-correlated with symptoms, although low forced expiratory volume in one second (FEV_1_) is associated with the risk of exacerbation^11–13^. Mostly, patients presented suboptimal asthma control but preserved FEV1^14^.

Oscillometry measures respiratory mechanics at different frequencies, such as airway resistance and reactance, to evaluate subtle changes in the small airway, inhomogeneity, and compliance of the peripheral lung. Performing oscillometry requires minimal effort and cooperation, which allows most patients to undertake the test, including children, elders, and people with disabilities^15–18^. Although exploring oscillometry is widely accepted in asthma diagnosis, symptom control, and phenotyping, the clinical significance and management of small airway dysfunction remain unclear and controversial^19–22^. There is no consensus on the routine detection of small airway abnormalities in patients with difficult-to-treat asthma. This study aimed to determine the potential oscillometry parameters that determine the difference between asthma patients with good or poor symptomatic control, focusing on those diagnosed with difficult-to-treat asthma.

## Methods

### Study population

**Figure 1:**
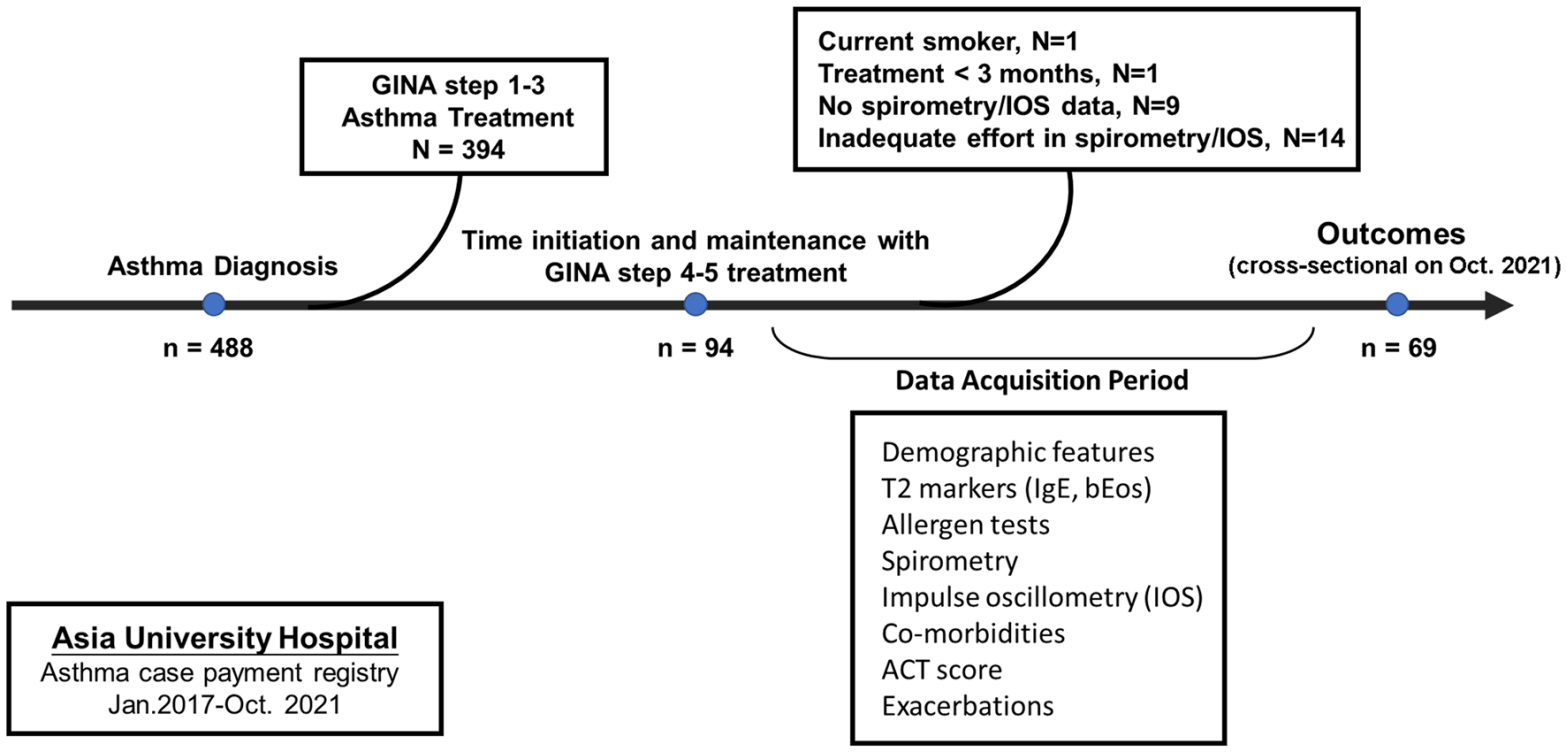
Flow chart for study population. *Abbreviation* ACT, Asthma Control Test; bEos, blood eosinophil level after asthma treatment with steps 4–5; GINA, Global Initiative for Asthma; IOS, impulse oscillometry system.

We conducted a cross-sectional study in October 2021 and retrospectively collected 488 patients between January 2017 and October 2021 from the asthma case payment registry at the Asia University Hospital. (Fig. 1.) The following patients were excluded from the study: patients who received GINA step 1–3 therapy (n = 394); patients who were currently smokers (n = 1); patients under GINA step 4–5 treatment for less than 3 months (n = 1); patients who were ineligible for spirometry and impulse oscillometry tests (n = 9), or had an inappropriate effort of spirometry and impulse oscillometry (n = 14). The final study population comprised of 69 patients with difficult-to-treat asthma. This study was conducted in accordance with the principles of the Declaration of Helsinki. The Institutional Review Board of Feng Yuan Hospital, Minister of Health and Welfare, approved our study (no.110016) and waived the requirement for informed consent.

### Assessment and data collection

Data regarding patient demographics, including age, sex, smoking history, medical treatment, immunological profiles (e.g., blood eosinophils before treatment and peak value during follow-up), fraction of exhaled nitrogen oxide (FeNO), serum IgE level, and allergen test results (Phadiatop and multiple antigen simultaneous tests) were collected from the electronic medical records. Spirometry and oscillometry data were obtained from patients who underwent GINA steps 4–5. We performed spirometry using MasterScreen Body/Diff (CareFusion, San Diego, CA, USA) and interpreted it according to the American Thoracic Society/European Respiratory Society guidelines^23,24^. We performed oscillometry using MasterScreen Impulse Oscillometry System (IOS; CareFusion, San Diego, CA, U.S.) and performed FeNO using NIOX VERO (Circassia, Oxford, U.K.) All procedures of spirometry and IOS fulfilled European Respiratory Society guidelines and we perform IOS before spirometry prior to and after administration of albuterol 400 mg, with acceptable test quality^16,23^.

### Outcomes

Outcome measurements were through asthma symptom control score and exacerbation calculated from at least 3 months of GINA steps 4–5 treatment. We used the asthma control test (ACT), a 5-item, patient-centered survey for assessing asthma control^25^, a cutoff score of 20 points to define patients with well-controlled or poorly controlled symptoms. Asthma exacerbation was classified with different degrees of severity based on electrical medical records, wherein: severe exacerbation is defined as emergency department visits or hospitalization requiring systemic corticosteroids or increasing dose from baseline; moderate exacerbation is defined as deterioration in the patient’s symptoms or lung function beyond day-to-day variations requiring a change of medication but that do not meet severe criteria^26,27^. Patients with at least one severe or two moderate exacerbations are defined as having frequent exacerbations.

### Statistical analyses

Categorical data from patients’ profiles were presented as numbers (%) and compared using Pearson’s chi-square test and Fisher’s exact test, as appropriate. Continuous variables are presented as means with standard deviation or median with interquartile range based on the Kolmogorov–Smirnov normality test, followed by Student’s t-test or the Mann–Whitney U test, respectively. Baseline characteristics of patients, spirometry, and IOS parameters were calculated using logistic regression for study endpoint analysis and multivariable analysis using significant variables (*P* < 0.20) in the multivariable logistic regression analysis. All tests were two-sided, and statistical significance was set at *P* < 0.05. All analyses were performed using SPSS software (version 25.0, IBM Corp., Chicago, IL, USA).

### Ethical approval

The Institutional Review Board of Feng Yuan Hospital, MOHW approved this study [111018].

## Results

Among the 69 patients enrolled, the median follow-up time was 1279 days. Of the total number of patients 49.3% were women, 65.2% were never-smokers, and 24 of 43 patients had positive allergen test results (26 patients didn’t perform allergen test). As for adjunctive therapy for asthma, all patients received long-acting muscarinic antagonists, 28 received leukotriene receptor antagonists, 16 received theophylline, and 14 received biological agents. Bronchodilator reversibility was observed in 16.1% of patients. Approximately 26.1% of patients experienced frequent exacerbations. Out of all the patients, 29.0% were diagnosed with chronic obstructive pulmonary disease (COPD), 26.9% with rhinosinusitis, and 14.5% with coronary artery disease. The demographic distribution of patients, categorized by an ACT score of 20 points, was not significantly different in terms of age, sex, medication, and other variables, but patients with poorly controlled asthma (ACT < 20) presented a higher prevalence of COPD (Table 1; see Supplementary Table 1 for detailed demographic profiles).

**Table 1:** Patient demographics and characteristics.

Characteristics	Total (n = 69)	ACT score		
		≥ 20 (n = 51)	< 20 (n = 18)	*P* value
Age at registry	62.0 [53.5,73.0]	62.0 [53.0,73.0]	67.0 [53.5,73.0]	0.547
Female	34/69 (49.3)	27 (52.9)	7 (38.9)	0.413
Former smoker	24/69 (34.8)	16 (31.4)	8 (44.4)	0.391
Post-Tx bEos (µL)	179.6 [88.4,325.3]	240.0 [105.6,335.2]	125.0 [61.3,196.3]	0.076
IgE (IU/mL)	77.6 [19.9,453.6]	120.7 [22.1, 475.2]	40.0 [10.8,471.6]	0.733
Comorbidity				
COPD	20 (29.0)	11 (21.6)	9 (50.0)	0.034
Bronchiectasis	5 (5.8%)	5 (9.8%)	0 (0.0)	0.316*
Allergic rhinitis/sinusitis	18 (26.1)	10 (19.6)	8 (44.4)	0.060*
CAD	10 (14.5)	6 (11.8)	4 (22.2)	0.436*
Reflux esophagitis	16 (23.2)	13 (25.5)	3 (16.7)	0.533*
Obstructive sleep Apnea	2 (2.9)	2(3.9)	0 (0.0)	1.000*
Treatment				
OCS	5 (7.2)	3 (5.9)	2 (11.1)	0.600*
Biologic agent	14 (20.3)	8 (15.7)	6 (33.3)	0.170*
Frequent exacerbation	18 (26.1)	13 (25.5)	5 (27.8)	1.000*
Spirometry				
PreBD FEV1 (L)	1.38 [0.98, 1.91]	1.38 [0.93,1.84]	1.44 [1.14, 2.04]	0.657
MMEF%pred (%)	32.0 [21.5, 45.5]	33.0 [20.0,47.0]	30.0 [22.0,43.5]	0.800
PostBD FEV_1_ (L)	1.43 [1.09,1.84]	1.40 [1.01,1.84]	1.53 [1.20,1.94]	0.552
PostBD FEV_1_/FVC (%)	70.7 [62.7,75.6]	71.3 [61.7,75.6]	70.1 [65.1,75.4	0.898
PostBD FEV1%pred (%)	68.5 [55.0,80.0]	67.0 [54.5,80.3]	70.0 [60.3,78.0]	0.664
Bronchodilator reversibility	10 (16.1)	6 (13.0)	4 (25.0)	0.266*

Categorical variables are presented as frequency (percentage) and compared with poorly controlled and well-controlled asthma using Pearson’s chi-square test and Fisher’s exact test. Continuous variables did not pass the Kolmogorov–Smirnov normality test and were recorded as median [interquartile range] and used a non-parametric test with the Mann–Whitney U Test.

ACT, asthma control test; AE, acute exacerbation; AR, allergic rhinitis; BMI, body mass index; Bronchodilator reversibility: positive with post-bronchodilation FEV1 change ≥  + 200 mL and change of predicted percentage ≥  + 12%; CAD, coronary artery disease; COPD, chronic obstructive pulmonary disease; CRS, chronic rhinosinusitis; FDR, frequency-dependent resistance; the difference in resistance at 5 Hz and 20 Hz; FEV_1_, forced expiratory volume in 1 s; F_res_, resonance frequency; FVC, forced vital capacity; IOS, impulse oscillometry; MMEF, maximal mid-expiratory flow; OCS, oral corticosteroid; Post-Tx bEos, blood eosinophil level after asthma treatment with steps 4–5 treatment.

*Fisher’s exact test.

**Exacerbation: Severe: Emergency visit or hospitalization; emergency, requiring systemic corticosteroids or increasing dose from baseline. Moderate: Deterioration in the patient’s symptoms or lung function beyond day-to-day variations requiring a change of medication but that does not meet severe criteria.

Frequent exacerbation: severe AE ≥ 1 time/year or moderate AE ≥ 2 times/year.

**Figure 2:**
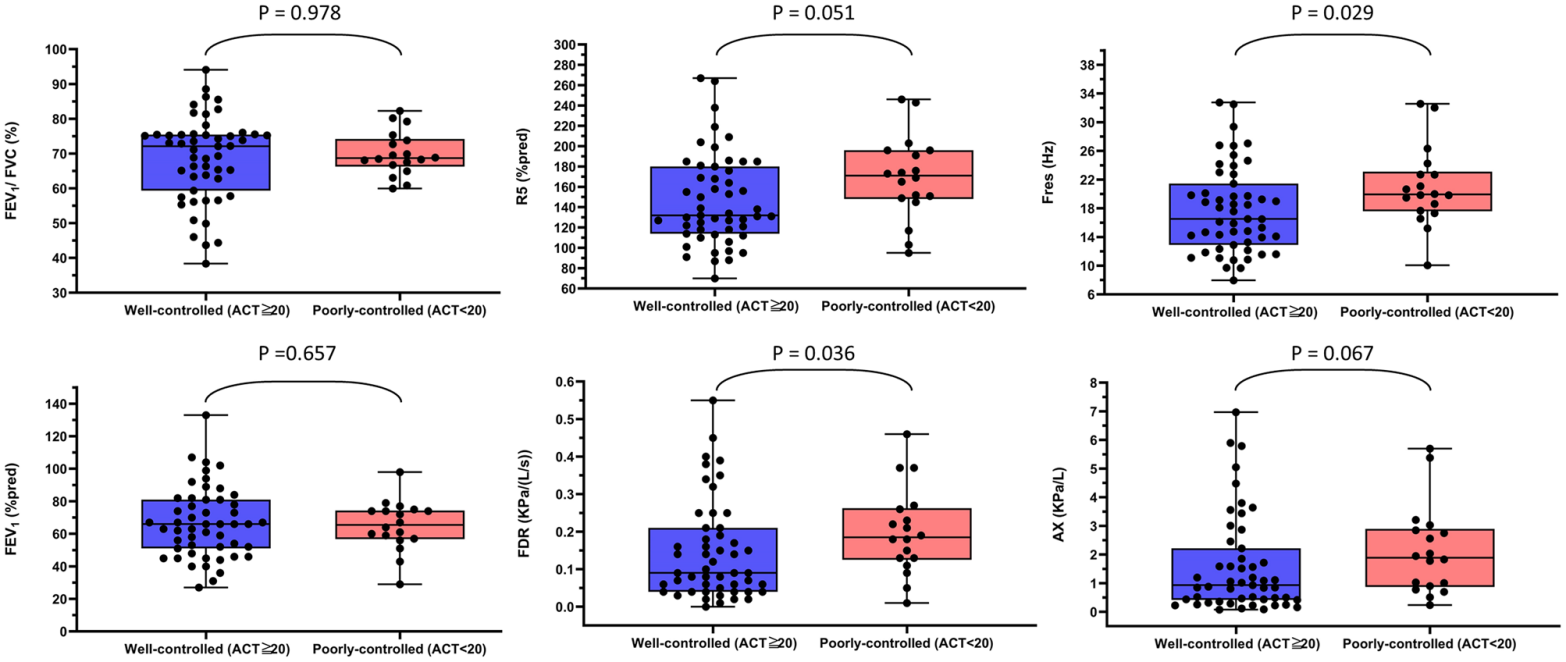
Differences in parameters of spirometry and IOS between well- and poorly-controlled asthma. *Abbreviation* ACT, asthma control test; AX, reactance area; FDR, frequency-dependent resistance; difference in resistance at 5 Hz and 20 Hz; FEV_1_, forced expiratory volume in 1 s; F_res_, resonance frequency; FVC, forced vital capacity; R_5_%pred, predicted percentage of resistance at 5 Hz.

Spirometry and IOS studies showed that the poorly controlled asthma group had significantly higher frequency-dependent resistance (FDR; difference in resistance at 5 Hz and 20 Hz) and resonance frequency (Fres) than the well-controlled asthma group (ACT ≥ 20) (Fig. 2). In the multivariable logistic regression model, higher FDR was an independent factor in increasing the odds ratio in poorly-controlled asthma (adjusted OR = 453.67, *P* = 0.043) and other independent factors: comorbidities with COPD (adjusted OR = 15.03, *P* = 0.003) and rhinosinusitis (adjusted OR = 11.16*, P* = 0.006) (Supplementary Table 2).

In the ROC curve analysis for predicting poorly controlled asthma, FDR demonstrated acceptable accuracy, with an area under the ROC curve of 66.7%, and a 95% confidence interval of 52.8–80.5%. The optimal cutoff FDR was 0.10, with a sensitivity of 83.3% and a specificity of 52.3% (Supplementary Figure 1). In the multivariable logistic regression model, patients with FDR ≥ 0.10 presented a higher strength of association with poorly controlled asthma than those with FDR < 0.10 (FDR ≥ 0.10 vs. < 0.10, adjusted ORR = 5.05, *P* = 0.037) (Table 2).

**Table 2:** Logistic regression model for poorly-controlled asthma (ACT < 20).

Variable	Univariate analysis		Multivariable analysis (*P* < 0.2)	
	OR (95% CI)	*P* value	aOR (95% CI)	*P* value
Post-Tx bEos (µL)	1.00 (0.99–1.00)	0.135	1.00 (0.99–1.00)	0.195
COPD (Yes vs. no)	3.55 (1.13–11.10)	0.030	10.35 (2.06–52.05)	0.005
CAD (Yes vs. no)	2.14 (0.53–8.69)	0.179		
AR/CRS (Yes vs. no)	3.28 (1.03–10.45)	0.044	8.56 (1.69–43.33)	0.009
FDR (kPa/(L/s)) (≥ 0.10 vs. < 0.10)	5.20 (1.34–20.17)	0.017	5.05 (1.10–23.21)	0.037

Eighteen patients with poorly controlled asthma were included in the study cohort. We selected variables with *P* < 0.2 in univariate analysis and then performed multivariable logistic regression analysis. (See detailed logistic regression analysis in Supplementary table 3).

AR, allergic rhinitis; CAD, coronary artery disease; COPD, chronic obstructive pulmonary disease; CRS, chronic rhinosinusitis; FDR, frequency-dependent resistance; the difference in resistance at 5 Hz and 20 Hz; F_res_, resonance frequency; OR, odds ratio; Post-tx bEos, blood eosinophil level after asthma treatment with steps 4–5 treatment.

**Figure 3:**
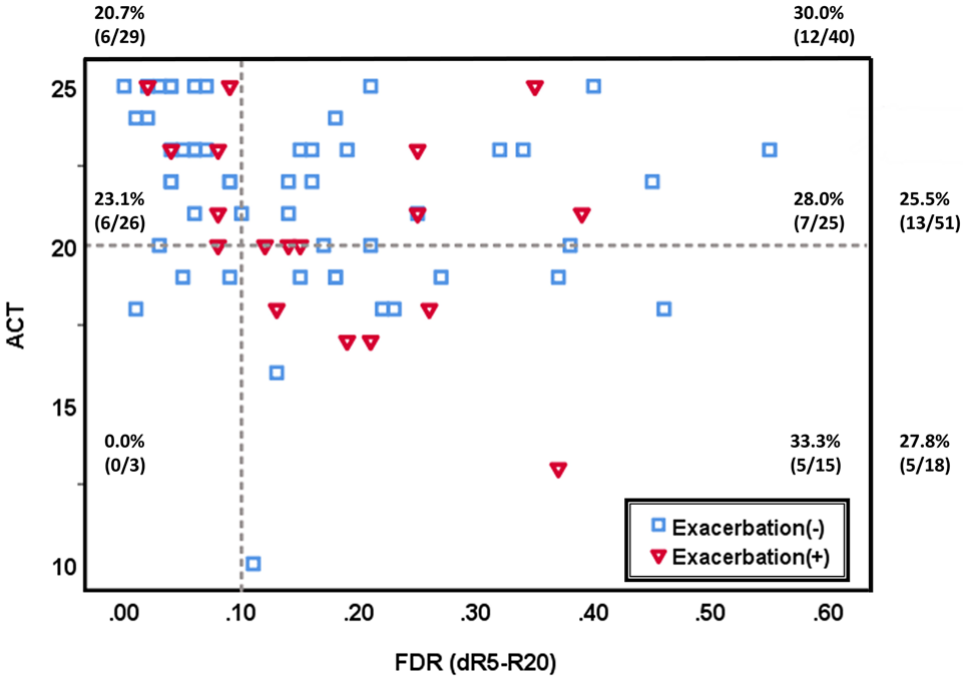
Distribution of ACT score, FDR, and frequent exacerbations in the study population. Based on the distribution of ACT and FDR, 30% of patients with FDR ≥ 0.10 (dashed line) experienced frequent exacerbations (once per year of severe acute exacerbation or twice per year of moderate acute exacerbation), and 27.8% of patients with ACT < 20 (dashed line) experienced frequent exacerbations. There were higher proportions of frequent exacerbations in patients with FDR ≥ 0.10 and ACT < 20, but the statistical difference in group comparisons was not met. *Abbreviation* ACT, asthma control test; FDR, frequency-dependent resistance; difference in resistance at 5 Hz and 20 Hz.

Figure 3 shows the distribution of ACT scores and FDR in our study samples, labeled according to the presence of frequent exacerbations in 12 months. There was a higher proportion of frequent exacerbations in patients with low ACT scores and high FDR than in the other three groups. However, no significant differences were observed. Moreover, the IOS parameters in our study failed to predict frequent exacerbations in the multivariable analysis. (Supplementary Table 3).

## Discussion

To date, there have been few studies on oscillometry parameters and asthma outcomes that have led to a similar conclusion^19,20,28,29^. Our study focused on evaluating oscillometry in patients with difficult-to-treat asthma and showed that FDR is an independent parameter associated with poor symptom control, irrespective of other comorbidities.

The goals of asthma management are to achieve optimum symptom control, reduce the future risk of exacerbations, optimize pulmonary functions, and minimize the adverse effects of medication. Assessment of clinical symptom scores is widely accepted in management of asthma. Previous studies used ACT as an appropriate measurement for various asthma outcomes^8^. However, we overestimated the clinical symptom scores of patients with poor perceptions or lack of knowledge regarding asthma control^30,31^. Clinical manifestations of asthma and the severity of airway inflammation remain imperfectly compatible. Pulmonary function is another aspect of clinical outcomes for the evaluation of airway remodeling. Previous reviews have claimed an association between spirometry and treatment outcome^11–13,32^; however, a discrepancy was still observed in several studies^9,33^. Patients with significant symptoms might have preserved FEV_1_ without fixed airway obstruction in real-world practice. Our results were consistent with those of previous studies and showed that the IOS parameters were more sensitive in predicting poor symptom control than spirometry in these patients^20^. Oscillometry requires minimal effort to perform and acts as an adjunctive tool for evaluating respiratory mechanics such as airway resistance and reactance. Regarding the instability of airway conditions in severe asthma patients, dynamic measurement of oscillometry parameters, including bronchodilator response and intrabreath difference, have proven to be effective in correlation to asthma outcomes^29,34,35^. Recent IOS studies have also explored the clinical utility of analyzing biological agents' therapeutic effects in severe asthma^36,37^. Pre- and post-treatment changes in IOS parameters could also indicate responsiveness^38^. Based on our findings and relevant studies, we suggest including oscillometry evaluation in the clinical assessment of difficult-to-treat asthma for the prognosis of symptom control and potential treatment responsiveness.

Although IOS parameters were associated with T2 inflammation and exacerbations in previous studies^34,39,40^, our data showed that IOS parameters did not significantly affect asthma exacerbations. Reports on performance variations among different devices should be taken into account. Previous studies have demonstrated variable respiratory impedance in the same model^41^. In the longitudinal outcomes of the ATLANTIC study, pre- and post-IOS parameter changes might predict the risk of exacerbation^28^. Moreover, lack of clinical data such as respiratory infection and allergen avoidance might also affect the data integrity of the analysis results.

However, our study has some limitations. First, it was a small, retrospective study with disproportional distributions of poorly- and well-controlled symptoms. Nonetheless, our findings are consistent with previous more extensive IOS studies, mainly focusing on patients undergoing GINA steps 4–5. Second, nearly 30% of patients had been diagnosed with COPD, and one-third of the patients were former smokers in our study population. The composition of patient demographics matches real-world practical scenarios rather than clinical trial settings. The selection bias between the exploration and management of comorbidities might have influenced clinical symptoms and treatment outcomes, such as pulmonary rehabilitation or consultation with other specialties. Despite these limitations, our study is representative of real-world experience. Further studies, in combination with imaging or pathology, are warranted to improve the clinical implications of IOS utility.

## Conclusion

In conclusion, our study implies that IOS is more sensitive than spirometry in predicting poor symptom control in patients with difficult-to-treat asthma under GINA steps 4–5. The application of IOS in these patients might help clinicians evaluate disease control and biologic treatment responsiveness, which can significantly improve asthma management.

## Supplementary Information

Supplementary Information.

## Data Availability

All data that support the findings of this study are available from the corresponding authors, Huang, upon reasonable request.
