# Supplementary material for: Assessing the prognostic value of respiratory oscillometry in patients with difficult-to-treat asthma
Source: Sci Rep. 2023 Feb 11;13:2457. doi: 10.1038/s41598-023-29672-z (PMC9922248; doi:10.1038/s41598-023-29672-z)
Supplement: Supplementary file 1 — Supplementary Information. [file 41598_2023_29672_MOESM1_ESM.docx]

**Title:**

**Assessing the prognostic value of respiratory oscillometry in patients with difficult-to-treat asthma**

**Authors:**

Yi-Luen Shen^1^, Yi-An Hsieh^1^, Yu-Ming Huang^2^, Yi-Hao Peng^2^, Ling-I Chen^1^, Fang-Chuan Dai^1^, Yu-Sheng Lin^1^, Chien-Wen Huang^1,3*^

**Author Affiliations:**

^1^Division of Chest Medicine, Department of Internal Medicine, Asia University Hospital, Taichung, Taiwan

^2^Department of Respiratory Therapy, Asia University Hospital, Taichung, Taiwan

^3^Department of Medical Laboratory Science and Biotechnology, College of Medical and Health Science, Asia University, Taichung, Taiwan

**^*^Corresponding Author:**

Chien-Wen Huang, MD, PhD

Division of Chest Medicine, Department of Internal Medicine, Asia University Hospital, Taichung, Taiwan

Address: No. 222, Fuxin Rd., Wufeng Dist., Taichung City 41354, Taiwan (R.O.C.)

Tel: 886-4-2332-9888

Fax:

E-mail: hjwn0403@gmail.com

**Supplementary Table 1. Demographics and characteristics of patients**

| **Characteristics** | **Total**  **(n=69)** |  | **ACT score** | | |
| --- | --- | --- | --- | --- | --- |
|  |  |  | **≥20 (n=51)** | **<20 (n=18)** | **P Value** |
| **Age at registry** | 62.0 [53.5, 73.0] |  | 62.0 [53.0, 73.0] | 67.0 [53.5, 73.0] | 0.547 |
| **Treatment duration (days)** | 817 [534, 1243] |  | 817 [535, 1245] | 798 [461, 1241] | 0.733 |
| **Follow-up time (days)** | 1279 [917, 1589] |  | 1311 [1008, 1588] | 1229 [652, 1591] | 0.428 |
| **Female** | 34/69 (49.3) |  | 27 (52.9) | 7 (38.9) | 0.413 |
| **Former smoker** | 24/69 (34.8) |  | 16 (31.4) | 8 (44.4) | 0.391 |
| **BMI (kg/m^2^)** | 25.4 [22.4, 28.7] |  | 25.7 [22.5, 29.3] | 24.2 [22.0, 28.4] | 0.571 |
| **Post-Tx bEos (uL)** | 179.6 [88.4, 325.3] |  | 240.0 [105.6,335.2] | 125.0 [61.3, 196.3] | 0.076 |
| **Peak bEos (uL)** | 321.7 [185.7, 487.4] |  | 328.3 [225.5,482.5] | 205 [104.7, 522.7] | 0.199 |
| **IgE (IU/mL)** | 77.6 [19.9, 453.6] |  | 120.7 [22.1, 475.2] | 40.0 [10.8, 471.6] | 0.733 |
| **Allergen test(+)** | 24/43 (55.8) |  | 17 (53.1) | 7 (63.6) | 0.728* |
| **Comorbidity** |  |  |  |  |  |
| **Hypertension** | 22 (31.9) |  | 16 (31.4) | 6 (33.3) | 1.000 |
| **Diabetes Mellitus** | 21 (30.4) |  | 17 (33.3) | 4 (22.0) | 0.553 |
| **COPD** | 20 (29.0) |  | 11 (21.6) | 9 (50.0) | 0.034 |
| **Bronchiectasis** | 5 (5.8%) |  | 5 (9.8%) | 0 (0.0) | 0.316* |
| **Allergic rhinitis/sinusitis** | 18 (26.1) |  | 10 (19.6) | 8 (44.4) | 0.060* |
| **Pulmonary TB** | 2 (2.9) |  | 2(3.9) | 0 (0.0) | 1.000* |
| **CAD** | 10 (14.5) |  | 6 (11.8) | 4 (22.2) | 0.436* |
| **Chronic Kidney disease** | 2 (2.9) |  | 2(3.9) | 0 (0.0) | 1.000* |
| **Reflux esophagitis** | 16 (23.2) |  | 13 (25.5) | 3 (16.7) | 0.533* |
| **Obstructive sleep Apnea** | 2 (2.9) |  | 2(3.9) | 0 (0.0) | 1.000* |
| **Treatment** |  |  |  |  | 1.000* |
| **MD-ICS** | 63 (91.3) |  | 47 (92.2) | 16 (88.9) |  |
| **HD-ICS** | 6 (8.7) |  | 4 (7.8) | 2 (11.1) |  |
| **Adjunct therapy** |  |  |  |  |  |
| **LAMA** | 69 (100.0) |  | 51 (100.0) | 18 (100.0) |  |
| **LTRA** | 28 (40.6) |  | 20 (39.2) | 8 (44.4) | 0.783 |
| **Theophylline** | 16 (23.2) |  | 12 (23.5) | 4 (22.2) | 1.000* |
| **OCS** | 5 (7.2) |  | 3 (5.9) | 2 (11.1) | 0.600* |
| **Open triple therapy** | 48 (69.6) |  | 34 (66.7) | 14 (77.8) | 0.553 |
| **Biologic agent** | 14 (20.3) |  | 8 (15.7) | 6 (33.3) | 0.170* |
| **Exacerbations**** |  |  |  |  |  |
| **Severe AE (time/yr)** | 0.23 (0-5) |  | 0.29 (0-5) | 0.06 (0-1) | 0.262 |
| **Moderate AE (time/yr)** | 0.51 (0-4) |  | 0.47 (0-3) | 0.61 (0-4) | 0.855 |
| **Frequent Exacerbation** | 18 (26.1) |  | 13 (25.5) | 5 (27.8) | 1.000* |
| **Spirometry** |  |  |  |  |  |
| **PreBD FVC (L)** | 2.11 [1.49, 2.68] |  | 1.83 [1.42, 2.74] | 2.16 [1.68, 2.71] | 0.687 |
| **PreBD FEV1 (L)** | 1.38 [0.98, 1.91] |  | 1.38 [0.93, 1.84] | 1.44 [1.14, 2.04] | 0.657 |
| **FEV1/FVC (%)** | 69.6 [63.2, 75.4] |  | 72.1 [59.4, 75.4] | 68.7 [66.3, 74.2] | 0.978 |
| **PreBD FEV1%pred (%)** | 66.0 [52.0, 77.5] |  | 66.0 [51.0, 81.0] | 65.5 [56.8, 74.3] | 0.973 |
| **MMEF%pred (%)** | 32.0 [21.5, 45.5] |  | 33.0 [20.0, 47.0] | 30.0 [22.0, 43.5] | 0.800 |
| **PostBD FVC (L)** | 2.12 [1.60, 2.75] |  | 2.10 [1.50, 2.83] | 2.12 [1.85, 2.70] | 0.562 |
| **PostBD FEV_1_ (L)** | 1.43 [1.09, 1.84] |  | 1.40 [1.01, 1.84] | 1.53 [1.20, 1.94] | 0.552 |
| **PostBD FEV_1_/FVC (%)** | 70.7 [62.7, 75.6] |  | 71.3 [61.7, 75.6] | 70.1 [65.1, 75.4 | 0.898 |
| **PostBD FEV1%pred (%)** | 68.5 [55.0, 80.0] |  | 67.0 [54.5, 80.3] | 70.0 [60.3, 78.0] | 0.664 |
| **Impulse Oscillometry (IOS)** |  |  |  |  |  |
| **R_5_%pred(%)** | 150.0 [118.0, 183.0] |  | 132.0 [114.0, 180.0] | 171.0 [148.0, 196.0] | 0.051 |
| **R_20_%pred(%)** | 127.0 [103.5, 147.0] |  | 124.0 [102.0, 142.0] | 132.5 [106.8, 150.8] | 0.318 |
| **FDR(kPa/(L/s))** | 0.14 [0.06, 0.23] |  | 0.09 [0.04, 0.21] | 0.19 [0.13, 0.26] | 0.036 |
| **X_5_(kPa/(L/s))** | -0.18 [-0.32, -0.13] |  | -0.17 [-0.32, -0.12] | -0.25 [-0.32, -0.15] | 0.194 |
| **Fres(Hz)** | 18.58 [14.16,22.71] |  | 16.52 [12.90, 21.43] | 19.93 [17.59, 23.10] | 0.029 |
| **AX(kPa/L)** | 1.06 [0.48,2.66] |  | 0.93 [0.42, 2.22] | 1.89 [0.93, 2.98] | 0.067 |
| **Coefficient 5 Hz** | 0.8 [0.7, 0.9] |  | 0.8 [0.7, 0.9] | 0.8 [0.6, 0.9] | 0.196 |
| **Coefficient 20 Hz** | 1.0 [0.9, 1.0] |  | 1.0 [0.9, 1.0] | 1.0 [0.9, 1.0] | 0.763 |
| **Bronchodilator Reversibility(BDR)** |  |  |  |  |  |
| **Spirometry BDR(+)** | 10 (16.1) |  | 6 (13.0) | 4 (25.0) | 0.266* |
| **IOS BDR(+)** |  |  |  |  |  |
| **R_5_%pred change ≥15%(+)** | 21 (37.5) |  | 14 (36.8) | 7 (38.9) | 1.000 |
| **FDR change ≥35%(+)** | 17 (30.4) |  | 10 (26.3) | 7 (38.9) | 0.366 |
| **X_5_ change ≥14%(+)** | 26 (46.4) |  | 16 (42.1) | 10 (55.6) | 0.399 |
| **AX change ≥35%(+)** | 21 (37.5) |  | 14 (36.8) | 7 (38.9) | 1.000 |
| **F_res_ change ≥15%(+)** | 22 (39.3) |  | 15 (39.5) | 7 (38.9) | 1.000 |

Categorical variables are presented as frequency (percentage) and compared with poorly controlled and well-controlled asthma using Pearson’s chi-square test and Fisher’s exact test. Continuous variables did not pass the Kolmogorov-Smirnov normality test and were recorded as median [interquartile range] and used a non-parametric test with the Mann-Whitney U Test.

***** Fisher’s exact test

** Exacerbation: Severe: Emergency visit or hospitalization; emergency, requiring systemic corticosteroids or increasing dose from baseline. Moderate: Deterioration in the patient’s symptoms or lung function beyond day-to-day variations requiring a change of medication but that does not meet severe criteria.

Frequent exacerbation: severe AE≥1 time/year or moderate AE ≥2 times/year.

**Abbreviation:** ACT: Asthma Control Test; AE: acute exacerbation; AR: allergic rhinitis; BMI: body mass index; Bronchodilator reversibility: positive with post-bronchodilation FEV1 change ≥ +200ml and change of predicted percentage ≥ +12%; CAD: coronary artery disease; COPD: chronic obstructive pulmonary disease; CRS: chronic rhinosinusitis; FDR: frequency-dependent resistance; the difference in resistance at 5Hz and 20 Hz; FEV_1_: forced expiratory volume in 1 s F_res_: resonance frequency; FVC: forced vital capacity; HD-ICS: inhaled corticosteroid dose equivalent to fluticasone propionate >500 $\mu$g/day; IOS: impulse oscillometry; LAMA: long-acting muscarinic antagonist; LTRA: leukotriene receptor antagonist; MD-ICS: inhaled corticosteroid dose equivalent to fluticasone propionate 250-500 $\mu$g/day; MMEF: maximal mid-expiratory flow; OCS: oral corticosteroid; Post-Tx bEos: blood eosinophils level after asthma treatment with step 4-5 treatment; R_5_%pred: predicted percentage of resistance at 5 Hz; R_20_%pred: predicted percentage of resistance at 20 Hz; ; X_5_: respiratory reactance at 5 Hz.

**Supplementary Figure 1. ROC Curve Analysis of FDR for poorly-controlled asthma**

**
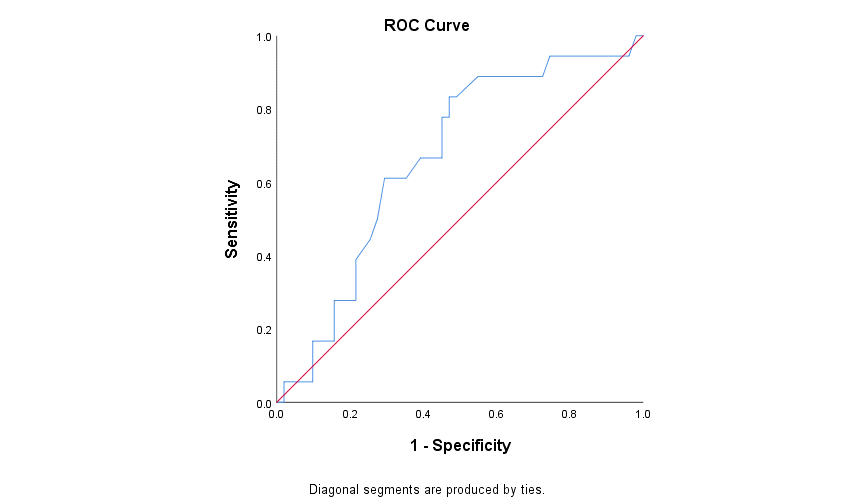

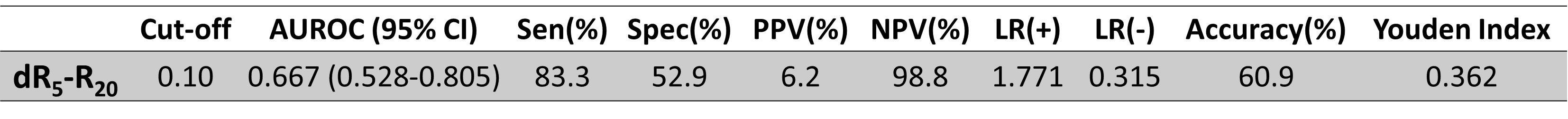
**

The area under receiver operating characteristic curve (AUROC) of difference of resistance at 5Hz and 20Hz(dR5-R20) level to discriminate ACT poor control after GINA step 4-5 treatments. The AUROC of dR5-R20 was 66.7% with 95% confidence interval of 52.8% to 80.5%. The optimal cut-off of dR5-R20 was 0.10 or less with a sensitivity of 83.3% and a specificity of 52.9%.

**Supplementary Table 2. Factors associated with frequent exacerbation (severe AE≥ 1 time/year or moderate AE≥ 2 time/year) (n =18)**

|  | **Univariate analysis** | |  | **Multivariable analysis (*P*<0.2)** | | | |
| --- | --- | --- | --- | --- | --- | --- | --- |
|  |  |  |  | **Model 1 (AX)** | | **Model 2 (X_5_)** | |
| **Variable** | **OR (95% CI)** | ***P* value** |  | **aOR (95% CI)** | ***P* value** | **aOR (95% CI)** | ***P* value** |
| **Gender (Female vs Male)** | 1.04 (0.36–3.05) | 0.943 |  |  |  |  |  |
| **BMI (kg/m^2^)** | 1.02 (0.94–1.11) | 0.625 |  |  |  |  |  |
| **Age at registry (years)** | 0.98 (0.94–1.02) | 0.365 |  |  |  |  |  |
| **Smoking (Yes vs. no)** | 1.27 (0.42–3.87) | 0.671 |  |  |  |  |  |
| **Post-Tx bEos (uL)** | 1.00 (0.99–1.00) | 0.252 |  |  |  |  |  |
| **Peak bEos (uL)** | 1.00 (1.00–1.00) | 0.121 |  | 1.00 (1.00-1.00) | 0.109 | 1.00 (1.00-1.00) | 0.108 |
| **IgE (IU)** | 1.00 (1.00–1.00) | 0.224 |  |  |  |  |  |
| **Detectable allergen (Yes vs. no)** | 0.86 (0.24–3.02) | 0.811 |  |  |  |  |  |
| **COPD (Yes vs. no)** | 1.29 (0.40–4.09) | 0.671 |  |  |  |  |  |
| **Bronchiectasis (Yes vs. no)** | 2.00 (0.31-13.06) | 0.469 |  |  |  |  |  |
| **Hypertension (Yes vs. no)** | 1.09 (0.35-3.44) | 0.878 |  |  |  |  |  |
| **CAD (Yes vs. no)** | 2.14 (0.53–8.69) | 0.286 |  |  |  |  |  |
| **AR/CRS (Yes vs. no)** | 0.76 (0.21–2.69) | 0.665 |  |  |  |  |  |
| **Diabetes Mellitus (Yes vs. no)** | 1.68 (0.54-5.20) | 0.367 |  |  |  |  |  |
| **Reflux esophagitis (Yes vs. no)** | 0.59 (0.15-2.35) | 0.449 |  |  |  |  |  |
| **HD-ICS vs MD-ICS** | 3.20 (0.58-17.55) | 0.180 |  |  |  |  |  |
| **Open triple vs Fix dose** | 4.75 (0.98-22.96) | 0.053 |  |  |  |  |  |
| **LTRA (yes vs no)** | 2.29 (0.77-6.84) | 0.137 |  |  |  |  |  |
| **Theophylline (yes vs no)** | 2.97 (0.90-9.76) | 0.073 |  |  |  |  |  |
| **PreFEV1 pred% (%)** | 1.00 (0.97-1.03) | 0.950 |  |  |  |  |  |
| **MMEF% (%)** | 1.01 (0.99-1.04) | 0.417 |  |  |  |  |  |
| **Spirometry BDR(+)** | 0.33 (0.04–2.89) | 0.319 |  |  |  |  |  |
| **R_5_pred% (%)** | 1.01 (1.00–1.02) | 0.257 |  |  |  |  |  |
| **FDR (kPa/(L/s))** | 4.07 (0.07-246.96) | 0.502 |  |  |  |  |  |
| **X_5_ (kPa/(L/s))** | 1.87 (0.61-56.96) | 0.720 |  |  |  |  |  |
| **AX (kPa/L)** | 1.03 (0.94-1.12) | 0.546 |  |  |  |  |  |
| **F_res_ (Hz)** | 1.02 (0.74–1.41) | 0.915 |  |  |  |  |  |
| **R_5_ %pred change ≥15%(+)** | 0.68 (0.20-2.34) | 0.542 |  |  |  |  |  |
| **FDR change ≥35%(+)** | 0.43 (0.10-1.76) | 0.240 |  |  |  |  |  |
| **X_5_ change ≥14%(+)** | 0.41 (0.12-1.40) | 0.155 |  |  |  | 0.43 (0.12-1.54) | 0.197 |
| **AX change ≥35%(+)** | 0.62 (0.18-2.10) | 0.438 |  |  |  |  |  |
| **F_res_ change ≥15%(+)** | 0.28 (0.07-1.12) | 0.077 |  | 0.27 (0.06-1.13) | 0.074 |  |  |

**Abbreviation:** ACT: Asthma Control Test; AE: acute exacerbation; aOR: adjusted odds ratio; AR: allergic rhinitis; BMI: body mass index; CAD: coronary artery disease; COPD: chronic obstructive pulmonary disease; CRS: chronic rhinosinusitis; FDR: frequency-dependent resistance; the difference in resistance at 5Hz and 20 Hz; FEV_1_: forced expiratory volume in 1 second; F_res_: resonance frequency; FVC: forced vital capacity; HD-ICS: inhaled corticosteroid dose equivalent to fluticasone propionate >500 mcg/day; IOS: impulse oscillometry; LAMA: long-acting muscarinic antagonist; LTRA: leukotriene receptor antagonist; MD-ICS: inhaled corticosteroid dose equivalent to fluticasone propionate 250-500 mcg/day; MMEF: maximal mid-expiratory flow; peak Eos: highest blood eosinophils level during whole cohort; OCS: oral corticosteroid; OR: odds ratio; Post-Tx bEos: blood eosinophils level after asthma treatment with step 4-5 treatment; R_5_%pred: predicted percentage of resistance at 5 Hz; Spirometry BDR: positive with post-bronchodilation FEV1 change ≥ +200ml and change of predicted percentage ≥ +12%; X_5_: respiratory reactance at 5 Hz.

**Supplementary Table 3. Factors associated with poorly-controlled asthma (ACT<20) (n =18)**

|  | **Univariate analysis** | |  | **Multivariable analysis (*P<0.2)*** | | | | | | | |
| --- | --- | --- | --- | --- | --- | --- | --- | --- | --- | --- | --- |
|  |  |  |  | **Model 1 (R_5_pred%)** | | | **Model 2 (FDR)** | | | **Model 3 (F_res_)** | |
| **Variable** | **OR (95% CI)** | ***P* value** |  | **aOR (95% CI)** | ***P* value** | **aOR (95% CI)** | | ***P* value** | **aOR (95% CI)** | | ***P* value** |
| **Gender (Female vs Male)** | 0.57 (0.19–1.69) | 0.308 |  |  |  |  | |  |  | |  |
| **BMI (kg/m^2^)** | 0.95 (0.86–1.06) | 0.355 |  |  |  |  | |  |  | |  |
| **Age at registry (years)** | 1.02 (0.97–1.06) | 0.477 |  |  |  |  | |  |  | |  |
| **Smoking (Yes vs. no)** | 1.75 (0.58–5.27) | 0.320 |  |  |  |  | |  |  | |  |
| **Post-Tx bEos (uL)** | 1.00 (0.99–1.00) | 0.135 |  | 1.00 (0.99-1.00) | 0.106 | 1.00 (0.99-1.00) | | 0.143 | 1.00 (0.99-1.00) | | 0.119 |
| **Peak bEos (uL)** | 1.00 (1.00–1.00) | 0.687 |  |  |  |  | |  |  | |  |
| **IgE (IU)** | 1.00 (1.00–1.00) | 0.243 |  |  |  |  | |  |  | |  |
| **Detectable allergen (Yes vs. no)** | 1.54 (0.38–6.33) | 0.546 |  |  |  |  | |  |  | |  |
| **COPD (Yes vs. no)** | 3.55 (1.13–11.10) | 0.030 |  | 13.30 (2.20-80.40) | 0.005 | 15.07 (2.49-91.17) | | 0.003 | 12.78 (2.17-75.36) | | 0.005 |
| **CAD (Yes vs. no)** | 2.14 (0.53–8.69) | 0.179 |  | 5.52 (0.70-43.28) | 0.104 |  | |  | 4.73 (0.58-38.42) | | 0.146 |
| **AR/CRS (Yes vs. no)** | 3.28 (1.03–10.45) | 0.044 |  | 14.88 (2.31-95.65) | 0.004 | 11.16 (2.01-61.80) | | 0.006 | 13.24 (2.11-82.95) | | 0.006 |
| **FEV1%pred (%)** | 1.00 (0.97-1.02) | 0.747 |  |  |  |  | |  |  | |  |
| **MMEF%pred (%)** | 1.00 (0.97-1.02) | 0.857 |  |  |  |  | |  |  | |  |
| **Spirometry BDR (Yes vs. no)** | 2.22 (0.54–9.20) | 0.270 |  |  |  |  | |  |  | |  |
| **R_5_pred% (%)** | 1.01 (1.00–1.02) | 0.073 |  | 1.01 (1.00-1.03) | 0.079 |  | |  |  | |  |
| **FDR (kPa/(L/s))** | 24.22 (0.40-1457.55) | 0.127 |  |  |  | 453.67 (1.22-169138.35) | | 0.043 |  | |  |
| **X_5_ (kPa/(L/s))** | 0.55 (0.02-13.67) | 0.716 |  |  |  |  | |  |  | |  |
| **AX (kPa/L)** | 1.20 (0.88-1.63) | 0.248 |  |  |  |  | |  |  | |  |
| **F_res_ (Hz)** | 1.09 (1.00–1.20) | 0.061 |  |  |  |  | |  | 1.08 (0.97-1.21) | | 0.152 |

**Abbreviation:** ACT: Asthma Control Test; AE: acute exacerbation; AR: allergic rhinitis; BMI: body mass index; aOR: adjusted odds ratio; CAD: coronary artery disease; COPD: chronic obstructive pulmonary disease; CRS: chronic rhinosinusitis; FDR: frequency-dependent resistance; the difference in resistance at 5Hz and 20 Hz; FEV_1_: forced expiratory volume in 1 second; F_res_: resonance frequency; MMEF: maximal mid-expiratory flow; peak Eos: highest blood eosinophils level during whole cohort; OR: odds ratio; Post-Tx bEos: blood eosinophils level after asthma treatment with step 4-5 treatment; R_5_%pred: predicted percentage of resistance at 5 Hz; Spirometry BDR: positive with post-bronchodilation FEV1 change ≥ +200ml and change of predicted percentage ≥ +12%; X_5_: respiratory reactance at 5 Hz.
